# Some Gastrointestinal Tract Characteristics of Karayaka Ram Lambs Slaughtered at Different Weights

**DOI:** 10.1155/2014/379023

**Published:** 2014-07-15

**Authors:** Arda Yıldırım, Yüksel Aksoy, Nuh Ocak, Zafer Ulutaş

**Affiliations:** ^1^Department of Animal Science, Faculty of Agriculture, University of Gaziosmanpaşa, 60240 Tokat, Turkey; ^2^Department of Food Processing, Bor Vocational School, University of Niğde, 51700 Niğde, Turkey; ^3^Department of Animal Science, Faculty of Agriculture, University of Ondokuz Mayıs, 55139 Samsun, Turkey; ^4^Department of Animal Production and Technologies, Faculty of Agricultural Sciences and Technologies, University of Niğde, 51240 Niğde, Turkey

## Abstract

Thirty-one Karayaka ram lambs were slaughtered at different body weights (30 (n = 7) , 35 (n = 6) , 40 (n = 7) , 45 (n = 6) , and 50 (n = 5) kg of body weight at fast) to evaluate the growth of their gastrointestinal tract (GIT) characteristics, to determine the relationship among slaughter body weight (SBW) and empty body weight (EBW), whole GIT and segments, and the influence of slaughter weight on the pH of rumen, jejunum, and cecal contents. The effects of the SBW on GIT weight (P < 0.05) , stomach ( P < 0.001) , and intestine (P < 0.05) , the body length (P < 0.001) and caecum (P < 0.05) , and the relative weights of GIT (P < 0.05) , stomach (P < 0.001) , and intestine (P < 0.001) were linear whereas that for the length of intestine were quadratic. The effect of SBW were quadratic (P < 0.05) on ratios of stomach to GIT weight and intestine length to intestine weight and rumen pH while, for the intestine to GIT weight ratio (P < 0.001) and caecum pH (P < 0.05) , this effect was linear. The results indicated that for all parameters studied, with the exception of intestinal length and cecal pH, linear relationships were observed with SBW indicating steady growth rates for these tissues.

## 1. Introduction

Gastrointestinal tract (GIT) development is characterized by tremendous increases in mass, volume, and surface area [1]. In addition, tissue growth is accompanied by a differentiation which results in physical changes which include increased GIT, stomach (reticulo-rumen), and intestinal capacity (relative to body weight) and alterations in metabolic characteristics. Rumen development has a clear and major impact on the digestive capabilities and supply of substrates to the growing ruminant [2]. Thus, understanding the control of growth and differentiation in the various segments' capacity of GIT is essential to evaluate the growth of noncarcass components of growing lambs and the development of improved feeding regimes and high carcass yield [3–5].

In meat production systems, an increase in slaughter weight of lambs may result in higher productivity and give more flexibility to the production system [6]. Traditionally, lambs in Turkey as well as in most parts of the world are slaughtered between weaning at approximately 3 to 6 months of age with slaughter weights of 10 to 12 kg for suckling lambs, 20 to 22 kg for light lambs, 30 to 32 kg for early fattening lambs, and 40 to 42 kg for late fattening lambs [7, 8]. As reported by Carvalho et al. [9] and Galvani et al. [10], proportions of different noncarcass components such as GIT, stomach, and intestine expressed as percentage of the body weight at slaughter and also the proportion of gastrointestinal content are the main factors that affect carcass yield. A high feed efficiency cannot always represent a high efficiency of food production, because some organs can be proportionally greater at more advanced maturity, dropping the retention of tissues in the carcass [11]. In order to achieve higher weight and quality carcass, necessary nutritional interventions are required in lamb from birth till slaughter [12]. Therefore, knowledge about growth rate from distinct body components can help to determine an adequate slaughter weight in which carcass yield will be maximized [10], whereas the study of growth rate of some organs with high metabolic activity including gastrointestinal organs can contribute to improving the understanding on factors that affect nutritional requirements of the animals [13].

Although a number of studies have been conducted to elucidate the GIT development and growth [7, 10, 14, 15], little information is available on the GIT characteristics and the influence of different slaughter weights on the pH values in the various segments (rumen, jejunum, and caecum) of GIT of especially the Karayaka ram lambs, which is one of the indigenous breeds reared on the coastline of the Black Sea region of Turkey and well adapted to the wet climate of the region. Thus, the GIT characteristics such as weights of whole GIT, stomach, and intestine, and lengths of body, intestine, and caecum should be studied at different slaughter weights. The present study was conducted to evaluate the growth of these GIT components of growing lambs, to provide increase in GIT and some segments which are closely related with the body weight of the animal, to determine the relationship among slaughter body weight (SBW) and empty body weight (EBW), whole GIT, and some segments; it is aimed to examine the influence of different slaughter weights on the pH values of the various segments (rumen, jejunum, and caecum) of the lamb GIT.

## 2. Material and Methods

The study was carried out at the experimental farm of the Gaziosmanpaşa University, Faculty of Agriculture, Tokat, Turkey, situated at 40°31′ N, 36°53′ E, and 650 m above sea level. Long term average annual temperature and relative humidity in this region vary from 8.1 to 14.2°C and between 56% and 73% [16]. The study was conducted complying with the EC 8 Directive 86/609/EEC and approved by Ethical Committee of Gaziosmanpaşa University for Experimental Animals (protocol number: 2011/046) and ascertained that the experiment is not an unnecessary repetition of previous experiments.

The winter born (*n* = 31) singleton indigenous Karayaka male lambs were used in this study. These animals were obtained from a study conducted to determine carcass and meat quality traits of Karayaka lambs at different slaughter weights in our institute [17]. Lambs were randomly assigned to one of five following slaughter weights: 30 (*n* = 7), 35 (*n* = 6), 40 (*n* = 7), 45 (*n* = 6), and 50 (*n* = 5) kg of body weight at fast. For this reason, lambs weaned at 42.3 ± 2.20 days of age were treated for internal parasites, drenched with anthelmintic preparation (Triclabendazole 12 mg/kg; Levamisole 7.5 mg/kg), and housed together in 4 × 6 m pens, equipped with feeders and a water source, and fed* ad libitum* under feedlot system in a naturally ventilated animal house.

Concentrate feed, mineral stone, and fresh water are given* ad libitum* in fattening period, whereas the forage lentil straw is given only 100 g/lamb/day during in the experiment. Feed was intended to provide nutrient requirements for fattening [18]. The chemical composition of the feed supplements is presented in Table 1. Dry matter (method 930.15), crude protein (method 954.01), and fat by ether extract were measured using the automated Tecator Soxtec System HT6 (Application note AN 301; FOSS North America, Eden Prairie, MN, USA), and crude ash (method 942.05) of dietary ingredients were determined according to AOAC [19]. The contents of neutral detergent fibre (NDF) and acid detergent fibre (ADF) were generated according to van Soest et al. [20] using the ANKOM A200/220 Fiber Analyzer filter bag technique (ANKOM Technology Corp., Fairport, NY, USA).

Before slaughter, the lambs were fasted for 16 h with free access to water. Also, body length (art. humeri-tuber ichii) was determined with measuring stick and measuring tape. When lambs reached 30, 35, 40, 45, and 50 kg of LW during fattening period, they were transported to a local licensed abattoir, and they were slaughtered following standard commercial slaughter procedures [21]. The digestive tract including the mouth, tongue, salivary glands, esophagus, four compartment stomach (rumen, reticulum, omasum, and abomasum), pancreas, gall bladder, small intestine (duodenum, jejunum, and ileum), and large intestine (caecum, colon, and rectum) was removed and weighed to get the weight of the full (GIT), and then emptied of its contents, washed, drained, and weighed to get the weight of the GIT content. GIT content was then subtracted from the SBW to determine empty body weight (EBW). Empty body weight (EBW) was calculated by the difference between SBW and the weight of the gastrointestinal contents [10]. Following exsanguinations, empty stomach (reticulo-rumen complex) and intestine weight were weighed and recorded [22]. As there is no distinct division between the rumen and the reticulum, they are often referred to together (reticulo-rumen). The rumen was opened, and the contents were removed before the organ was washed by hand and the external fat was then removed [23]. Length of the intestine was measured with measuring stick and measuring tape [24]. Empty GIT, stomach, and intestine weights were weighed (absolute weight) and expressed (relative weight, % of SBW) per unit of body weight at slaughter. Hence, the relative weight contributions of these non-carcass components to SBW or their commercial yields were determined.

Before evisceration, the pH values of rumen, jejunum, and caecum digestas were immediately determined using a digital pH meter (Sartorious PP15, AG Weender Landstrasse 94–108, Goettingen, Germany). In the present study, the pH values were determined in five slaughter weights of rumen, jejunum, and caecum because rumen and intestine digesta pH values are dependent on sampling location within rumen and intestine. The pH probe was kept in the rumen, duodenum, and caecum until the pH reading was stabilized, and pH value was recorded [23].

Data were analyzed in a completely randomized design by regression. Thus, model included linear and quadratic effects as well as the lone main effect of slaughter weight, using the obtained values as independent variables. All analyses were performed using the SPSS statistical package [25]. Regression equations and bivariate correlations displaying the relationship among slaughter weight and weights of GIT, stomach, and intestine and lengths of body, intestine, and caecum were to be examined by means of regression procedure of SPSS. To verify if *b* is significant, the *t*-test was used. Results are presented as means and a pooled SEM (unless otherwise stated).

## 3. Results 

The SBW, EBW, the absolute weights and length, and the relative weights of whole GIT and their segments of Karayaka ram lambs are presented in Table 2. The effects of the SBW for the absolute weights of GIT (*P* < 0.05), stomach (*P* < 0.001), and intestine (*P* < 0.05), the lengths of body (*P* < 0.001) and caecum (*P* < 0.05), and the relative weights of GIT (*P* < 0.05), stomach (*P* < 0.001), intestine (*P* < 0.001), and the gastrointestinal content (*P* < 0.05) were linear whereas those for the absolute weight of gastrointestinal content (*P* < 0.05) and the length of intestine were quadratic.

Estimated parameters for the regression equations of the empty body weight, whole GIT, and their segments and bivariate correlations displaying the relationship among these variables and slaughter weight are presented in Table 3. The relationships between the SBW and EBW (*r* ^2^ = 0.986, *P* < 0.001) and the weights of GIT (*r* ^2^ = 0.329, *P* < 0.01) and stomach (*r* ^2^ = 0.512, *P* < 0.01) and the length of body (*r* ^2^ = 0.376, *P* < 0.01) and caecum (*r* ^2^ = 0.145, *P* < 0.05) were significant. The relationship between the EBW and SBW can be expressed by the following linear equation (*r* ^2^ = 0.986; standard error of the estimate = 0.813, *n* = 31): EBW, kg = −1.81 (±0.818) + [0.968 (±0.200) × SBW, kg].

The intestine length to body length ratio, stomach weight to gastrointestinal weight ratio, intestine weight to gastrointestinal weight ratio, and intestine length to intestine weight ratio in Karayaka ram lambs slaughtered at different weights are presented in Table 4. The effects of the SBW were quadratic (*P* < 0.05) for the ratios of stomach weight to GIT weight and intestine length to intestine weight, while for theintestine weight to GIT weight ratio (*P* < 0.001), this effect was linear.

The pH of contents from various segments of the GIT is presented in Table 5. The effects of the SBW were linear (*P* < 0.05) for the rumen pH while for the caecum pH (*P* < 0.05), these effects were quadratic. A search of the literature indicated a lack of information on pH values of the various segments of the GIT in lambs slaughtered at different body weights.

## 4. Discussion

In general, a majority of the parameters measured exhibited linear relationships with SBW, with the exception of digesta and length and cecal pH which exhibited a quadratic relationship. These results confirm that the highest carcass yield obtained from lambs slaughtered immediately after weaning [15, 26] although changes in the carcass yield as the SBW increased were not presented in the current study. The absolute weights of whole GIT and stomach increased linearly whereas that of intestine decreased as the SBW increased. The functional maturity of the GIT is essential for the survival and growth of postnatal animals [1]. Therefore, the growth patterns of different body components such as whole GIT, stomach, and intestines in relation to SBW were decreased linearly as the SBW increased. The relative weights of noncarcass components were greater in lambs slaughtered at 30 and 35 kg of body weight than those in lambs slaughtered at 40, 45, and 50 kg of body weight. These results indicate that the changes in the relative weight contributions of noncarcass components to SBW affected the SBW or commercial carcass yields.

In the study reported by Abdullah and Qudsieh [7], slaughtering Awassi ram lambs at weights up to 30 kg resulted in higher dressing-out percentage and better carcass characteristics than ram lambs slaughtered at heavier weights. Lambs with heavier weights of stomach and intestine may have lower carcass yield because the gastrointestinal content influenced carcass yield [4]. Therefore, when taking account of the change in GIT weight with increasing SBW and the quadratic change in contents, the optimal body weight for slaughter may be recommended as 40–45 kg for Karayaka male lambs. Indeed the gastrointestinal content as a proportion of SBW, which indicates the ratio of EBW to SBW, decreased from 8.9% in lambs slaughtered at body weight of 30 kg to 7.0% in animals slaughtered at more elevated body weights. This may be explained by the fact that the proportion of gastrointestinal content is directly related to the ruminal development [10], which is stimulated by the dry matter intake, mainly from fibrous foods [2]. Accordingly, for Karayaka lambs raised in confinement after weaning at 42 days and feeding until 150 days of age, slaughter weight targets should probably be increased to 40–45 kg without detrimental effects on carcass yield [17], confirming the suggestions of Santos-Silva and Portugal [27] for Merino Branco lambs. The results on the absolute weights and length and the relative weights of whole GIT and their segments are similar to those reported by Galvani et al. [10] in Texel crossbred lambs slaughtered at 25, 30, or 35 kg of body weight. Indeed, Galvani et al. [10] reported that an effect of the SBW on the absolute weights and relative weights of GIT, stomach, and intestine was observed while there is no effect on weight of stomach contents. Also, Balci and Karakas [14] found that effect of the SBW on the absolute weight of GIT in Karayaka ram lambs slaughtered at 25, 30, or 35 kg of body weight was significant.

The development of stomach is directly related to feed intake [2]. Priolo et al. [28] reported that the increase in weight of the rumen and small intestine is an indication that they have an important role in ensuring the efficiency of feed utilization in animals. However, it was not clear whether lambs slaughtered at a high body weight were more efficient in terms of feed intake and feed efficiency due to the difference in relative GIT weights since there were no data on the amount of feed consumed by lambs in the current study. In the study, there is the linear decrease in the absolute and relative weights of intestine while there is a quadratic change in the length of intestine (Table 3). Therefore, the proportion of intestine was affected by the slaughter weight, as reported by Galvani et al. [10].

Correlation coefficients found in the present study are lower than those observed by Galvani et al. [10] for the small and large intestines; our results may be analyzed by using the regression equations of each of the GIT segments, as a function of SBW, with the exception of intestine weight and length. In the present study, the EBW, the weights of GIT and their segments such as stomach and intestine caecum increased at a steady rate between all slaughter weights, as reported by Balci and Karakas [14], Abdullah and Qudsieh [7], and Galvani et al. [10].

The results with respect to the ratios between the whole GIT traits and their segments indicate that the ratios of stomach weight to GIT weight and intestine length to intestine weight and the intestine weight to GIT weight are likely to affect the fattening performance of lambs, as it was observed in our previous study [29]. Also, these results may suggest that different parts of the GIT had a constant length relative to the intestine weight. The quadratic effect of the SBW for the ratios of intestine length to intestine weight may be explained by the fact that stimulating the functional role of the GIT at an early age seems to increase its length [5].

The pH values observed in the present study were within ranges (6.00 to 7.08) which most rumen microbes can typically thrive [29–31]. The GIT development may be stimulated by pH values of rumen and caecum because volatile fatty acids may result in significant changes in intestinal growth characteristics of developing ruminants [1]. Ruminal pH drops below physiological levels when ruminants consume excessive amounts of rapidly fermentable (nonfibre) carbohydrates [32, 33]. Krause and Oetzel [33] noted that, to maintain ruminal pH within a physiological range of about 5.5 to 7.0, ruminants possess highly developed systems such as carefully regulating their feed intake and the ability of the rumen to rapidly absorb organic acids. Therefore, the differences in influence of SBW on the GIT pH may be explained by differences in the nature of regulation of ruminal pH described above.

## 5. Conclusions

The results indicated that, for all parameters studied, except gastrointestinal content, intestine length and caecum pH increased at a steady rate between all slaughter weights and also that different body components present distinct growth patterns; GIT, stomach, and intestine are, proportionally, greater in the younger Karayaka sheep. Also, growth and development of whole GIT and specific segments increased with SBW, and it is evident that they was not reached a plateau for except intestine length. The changes in the relative weights of the whole GIT and their segments as well as the relative weight of the gastrointestinal content as the SBW increased suggested that 40 and 45 kg of slaughter weight for Karayaka male lambs are recommended. However, because the response in terms of carcass yield and quality parameters can vary depending on the degree of maturity of the animals at slaughter, the age or body weight of the lamb at slaughter should be determined. However, because the response in terms of carcass yield and quality parameters can vary depending on the degree of maturity of the animals at slaughter, the age or body weight of the lamb at slaughter should be determined taking into account demands and prices for these carcass components in different markets. These data may be used in selection of optimal slaughter weight decisions on farm regarding Karayaka ram lambs.

## Figures and Tables

**Table 1:** Composition of experimental concentrate feed and red lentil straw.

Item	Concentrate feed	Red lentil straw
Metabolizable energy (Mcal/kg)	2.77	2.01
Nutrients (%)		
Dry matter	92.00	91.30
Crude protein	20.63	5.78
Ether extract	2.60	1.49
Ash	10.40	9.60
Neutral detergent fibre	37.96	56.29
Acid detergent fibre	26.39	55.59

**Table 2:** Slaughter body (SBW) and empty body (EBW) weights (kg), absolute weights (kg) and length (m), and relative weights (% of SBW) of gastrointestinal tract segments of Karayaka ram lambs.

Variable	Slaughter weight categories (kg)					SEM^(1)^	*P*	
	30 (*n* = 7)	35 (*n* = 6)	40 (*n* = 7)	45 (*n* = 6)	50 (*n* = 5)		*L* ^(2)^	*Q* ^(3)^
Weight of								
Slaughter body	30.1	35.6	40.3	45.1	50.6	1.23		
Empty body	27.4	32.4	37.4	41.8	47.1	1.19	∗∗∗	NS
Gastrointestinal tract	5.76	6.34	6.86	6.89	7.60	0.200	∗	NS
Stomach	0.82	0.86	0.91	0.95	1.06	0.019	∗∗∗	NS
Intestine	1.12	1.04	1.04	1.03	1.06	0.022	∗	NS
Gastrointestinal content	2.70	3.18	2.90	3.28	3.52	0.145	NS	∗
Length of								
Body	0.59	0.60	0.64	0.63	0.68	0.008	∗∗∗	NS
Intestine	34.6	34.2	37.8	35.1	35.4	0.44	NS	∗
Caecum	0.34	0.38	0.40	0.37	0.43	0.010	∗	NS
Relative weight of								
Gastrointestinal tract	19.1	17.8	17.0	15.3	15.0	0.48	∗	NS
Stomach	2.7	2.4	2.3	2.1	2.1	0.05	∗∗∗	NS
Intestine	3.7	2.9	2.6	2.1	2.1	0.12	∗∗∗	NS
Gastrointestinal content	8.9	8.9	7.2	7.3	7.0	0.39	∗	NS

^(1)^Standard error of the mean; ^(2)^linear and ^(3)^quadratic effects of increasing slaughter weight; NS: nonsignificant (*P* > 0.05); *P < 0.05; **P < 0.01; ***P < 0.001.

**Table 3:** Estimated parameters for the regression equations of the empty body weight (EBW), whole gastrointestinal tract (GIT), and some segments of GIT and bivariate correlations displaying the relationship among these variables and slaughter weight.

Component	*a*	*b*	SE^†^	*t*	*r* ^2^	SE^§^
EBW	−1.808	0.968	0.200	47.374∗∗∗	0.986∗∗∗	0.813
GIT weight	2.934	0.093	0.024	3.888∗∗∗	0.329∗∗	0.957
Stomach weight	0.463	0.011	0.002	5.707∗∗∗	0.512∗∗	0.079
Intestine weight	1.215	−0.004	0.003	−1.444^NS^	0.063^NS^	0.123
Body length	47.103	0.390	0.090	13.033∗∗∗	0.376∗∗	3.594
Intestine length	32.561	0.072	0.060	1.195^NS^	0.044^NS^	2.407
Caecum length	25.258	0.321	0.142	2.257∗	0.145∗	5.462

*a*: the intercept of the linear regression; *b*: the relative growth or regression coefficient; SE^†^: standard error of the regression coefficient, SE^§^: standard error of the estimate, ^NS^ *P* > 0.05; *P < 0.05; **P < 0.01; ***P < 0.001.

**Table 4:** Ratios between the whole GIT and selected segments in the Karayaka ram lambs slaughtered at different weights.

Variable	Slaughter weight categories (kg)					SEM^(1)^	*P*	
	30 (*n* = 7)	35 (*n* = 6)	40 (*n* = 7)	45 (*n* = 6)	50 (*n* = 5)		*L* ^(2)^	*Q* ^(3)^
IL to BL	58.7	57.6	58.7	55.9	52.3	0.97	NS	NS
SW to GITW	14.5	13.6	13.6	14.0	14.2	0.36	NS	∗
IW to GITW	19.8	16.3	15.5	13.8	14.4	0.58	∗∗	NS
IL to IW	3.2	3.0	2.8	2.7	3.0	0.06	NS	∗

IL to BL: intestine length to body length ratio; SW to GITW: stomach weight to gastrointestinal weight ratio; IW to GITW: intestine weight to gastrointestinal weight ratio; IL to IW: intestine length to intestine weight ratio.

^(1)^Standard error of the mean; ^(2)^linear and ^(3)^quadratic effects of increasing slaughter weight; NS: nonsignificant (*P* > 0.05); *P < 0.05; **P < 0.01.

**Table 5:** Selected segment pH of gastrointestinal tract in Karayaka ram lambs slaughtered at different weights.

Variable	Slaughter weight categories (kg)					SEM^(1)^	*P*	
	30 (*n* = 7)	35 (*n* = 6)	40 (*n* = 7)	45 (*n* = 6)	50 (*n* = 5)		*L* ^(2)^	*Q* ^(3)^
Rumen pH	6.87	6.91	6.98	6.84	7.08	0.419	∗	NS
Jejunum pH	6.00	6.05	6.09	6.01	6.06	0.602	NS	NS
Caecum pH	6.47	6.59	6.77	6.64	6.47	0.036	NS	∗

^(1)^Standard error of the mean; ^(2)^linear and ^(3)^quadratic effects of increasing slaughter weight; NS: nonsignificant (*P* > 0.05); *P < 0.05.
